# The relationship of comorbidities to mortality and cause of death in patients with differentiated thyroid carcinoma

**DOI:** 10.1038/s41598-019-47898-8

**Published:** 2019-08-07

**Authors:** Young Ki Lee, Namki Hong, Se Hee Park, Dong Yeob Shin, Cho Rok Lee, Sang-Wook Kang, Jandee Lee, Jong Ju Jeong, Kee-Hyun Nam, Woong Youn Chung, Eun Jig Lee

**Affiliations:** 1 Division of Endocrinology and Metabolism, Department of Internal Medicine, Yonsei University College of Medicine, Seoul, 03722 Republic of Korea; 2 Center for Thyroid Cancer, National Cancer center, Goyang, 10408 Republic of Korea; 3 Division of Endocrinology and Metabolism, Department of Internal Medicine, National Health Insurance Service Ilsan Hospital, Goyang, 10444 Republic of Korea; 4 Division of Thyroid and Endocrine Surgery, Department of Surgery, Yonsei University College of Medicine, Seoul, 03722 Republic of Korea

**Keywords:** Head and neck cancer, Thyroid diseases

## Abstract

Understanding how comorbidities contribute to death in cancer patients is becoming an important topic. The present study assessed the role of comorbidities in overall mortality and causes of death in patients with differentiated thyroid carcinoma (DTC). This retrospective cohort study included 2070 patients who underwent thyroidectomy for DTC at a single institution between 2002 and 2005. Probabilities of overall, DTC-specific and other-cause death were examined according to the number of comorbidities, with consideration for competing events. The estimated 15-year cumulative incidences of overall, DTC-specific, and other-cause death were 7.3%, 1.6%, and 5.7%, respectively. Taking the group without comorbidities as a reference, we found that the group with 1–2 comorbidities and the group with ≥3 comorbidities had higher probabilities of other-cause death (subhazard ratios = 2.48 and 9.41, respectively; p < 0.01) and consequently shorter overall survival (hazard ratio = 1.95 and 5.33, respectively; p < 0.01), with adjustment for age, sex, and tumor-node-metastasis classification. In contrast, the probability of DTC-specific death was reduced in patients with ≥3 comorbidities (subhazard ratio = 6.81e-10, p < 0.01). For overall death, the relative proportion of death from DTC reduced when the number of comorbidities increased, and DTC-specific death was not observed in patients with ≥3 comorbidities. Our results show that death from DTC itself accounted for only a fraction of the overall deaths among patients who underwent surgery for DTC. Comorbidities increased overall mortality by increasing the probability of other-cause death. Patients with multiple comorbidities had a low probability of dying from DTC because they died earlier from comorbidities.

## Introduction

Comorbidities are coexisting medical conditions in a patient who has an index disease of interest^1^. Patients with cancer are often diagnosed with comorbidities that can reduce their overall survival^2,3^. Comorbidities are known to increase the rates of non-cancer deaths (death from non-cancer causes in patients with cancer) and have a greater impact on the survival of patients with less severe cancers^2^.

Differentiated thyroid carcinoma (DTC) is understood to be relatively indolent. The majority of patients with DTC are in stage I or II according to the American Joint Committee on Cancer/Union for International Cancer Control (AJCC/UICC) staging system, with a 10-year disease-specific survival of more than 90%^4–7^. However, the overall survival of patients with DTC is significantly lower than disease-specific survival, and the 10-year overall survival of patients in AJCC/UICC stage II was reported to be only 71%^5^. This observation suggests that there may be a large contribution of comorbidities toward the death of patients with DTC. Nevertheless, little is known about the role of comorbidities in leading to death in patients with DTC. The aim of this study is to evaluate the predictive ability of comorbidities at the time of surgery for the overall mortality and the cause of death in patients with DTC.

## Materials and Methods

### Patients

We performed a single-center, retrospective cohort study that included patients who underwent thyroidectomy (total thyroidectomy or hemi-thyroidectomy) for DTC between 2002 and 2005 at Severance Hospital in Seoul, Republic of Korea. Only patients with pathologically proven papillary thyroid carcinoma, follicular thyroid carcinoma, and Hurthle cell thyroid carcinoma were included in this study; patients with other types of thyroid cancer were excluded. In addition, patients with noninvasive follicular thyroid neoplasm with papillary-like nuclear features (NIFTP) or those with papillary thyroid microcarcinoma (size < 1 cm) without lymph node metastases or distant metastases were excluded to minimize the influence of data from patients with tumors carrying minimal expected disease-specific mortality.

### Classifications for thyroid cancer, comorbidities, and outcomes

The medical records of all patients were manually reviewed. Patients were retrospectively reclassified according to the eighth edition of AJCC/UICC tumor-node-metastasis (TNM) classification system^8^. For all patients, history taking and routine workup (e.g., electrocardiogram, chest radiograph, serum glucose, creatinine, aspartate transaminase, alanine transaminase, and total bilirubin) were thoroughly performed before surgery to assess operability. Patients with a history of comorbidities or suspected comorbidities found during the routine workup were referred to relevant specialists; additional examinations (e.g., echocardiography, coronary angiography, pulmonary function test) and treatments were conducted as needed before surgery.

Diseases mentioned in the patient’s past history or diagnosed during preoperative workup were considered to be baseline comorbidities. We identified baseline comorbidities as follows: malignancies other than DTC, hypertension (HTN), acute coronary syndrome (myocardial infarction and unstable angina), coronary artery occlusive disease (coronary artery disease proven through coronary angiography, as well as acute coronary syndrome), congestive heart failure, peripheral vascular disease, cerebrovascular accident, valvular heart disease (grade III and IV), aortic dissection, atrial fibrillation, diabetes mellitus (DM), chronic viral hepatitis, liver cirrhosis, asthma, chronic obstructive lung disease, end-stage renal disease, connective tissue disease, dementia, plegia (hemiplegia, paraplegia, and quadriplegia), peptic ulcer disease, and acquired immunodeficiency syndrome (AIDS). Most of these baseline comorbidities were comorbid conditions described by Charlson *et al*.^9^.

Survival status during the follow-up period through March 5, 2018 was assessed. To determine survival status and cause of death, we additionally reviewed the in-hospital survival-database, which has been built for appropriate medical care and follow-up. The in-hospital survival-database included records of telephonic interviews with patients or family members of patients, patients’ qualification for the National Health Insurance (NHI) of the Republic of Korea, as well as the date and cause of death of patients according to the Korean Central Cancer Registry (KCCR) database. The NHI system of the Republic of Korea is a mandatory universal health insurance system that covers the entire Korean population. On death, patients automatically lose qualification for the NHI. The KCCR was started by the Ministry of Health and Welfare in the Republic of Korea in 1980 and then expanded nationwide; it covers more than 95% of cancer patients in the Republic of Korea^10^. Patients with valid qualification for the NHI were classified as alive. If a patient was not qualified for the NHI but their death was not proven by any of the medical records, records of telephonic interviews, or the KCCR database, the patient’s data were considered to be censored at the time of the last follow-up; this situation could occur if the patient was a foreigner, was of lost Korean nationality, had immigrated, or if a record of the patient’s death was simply missing from the database. Deaths were classified as death from DTC or death from other causes. Death from DTC was defined as a case where DTC was the primary cause of death or a complication from DTC was direct cause of death in the presence of DTC progression (e.g., death from pneumonia associated with the progression of lung metastasis from DTC). Death from other causes was defined as a case in which the primary cause of death was something other than DTC or in which no evidence of residual thyroid cancer was observed at the time of death.

### Survival analyses for overall mortality

The Kaplan-Meier method was used to generate survival curves according to the number of comorbidities for each patient (0, 1–2, and ≥3), while the log-rank test was performed to evaluate differences in overall survival. The combined effects of the T, N, and M classifications of DTC and number of comorbidities on overall survival were evaluated using a multivariable Cox proportional hazard model with adjustment for age (<55, 55–65, and ≥65 years), sex, and histological type. The validity of the proportional hazards assumption in the Cox model was confirmed using Schoenfeld residuals and parallel curves of the log-minus-log plots. The hazard ratios (HR) for overall survival are presented with 95% confidence intervals (CIs).

### Survival analyses with consideration for competing events

Cumulative incidence plots were generated for death from DTC and death from other causes with the competing-risks method^11,12^. The multivariable Fine and Gray’s regression model was used to assess the effect of T, N, and M classifications of DTC and the number of comorbidities on death from DTC and death from other causes in the presence of competing risks^13^. The subhazard ratios (SHR) for death from DTC and death from other causes are presented with 95% CIs. SHR estimates were adjusted for variables that were statistically significant in the preceding multivariable Cox proportional hazard model for overall survival. For generalization, we also performed multivariable Fine and Gray’s regression analysis using the Charlson comorbidity index (age and thyroid cancer were not included in the calculation of the index), instead of the number of comorbidities^9^. Additionally, stratified cumulative incidence plots for death from DTC and death from other causes were generated according to AJCC/UICC TNM stages and the number of comorbidities.

### Statistical analyses

Descriptive statistics were presented as means ± standard deviations, medians (interquartile range), and numbers (%) depending on the type of variables.

All tests were two-sided. A *P* value of <0.05 was considered to be statistically significant. All statistical analyses were performed using SPSS version 23.0 for Windows (IBM Corporation, Armonk, NY, USA) or STATA 14.0 (Stata Corporation, College Station, TX, USA).

### Ethics statement

This study was approved by the institutional review board of Severance Hospital, Yonsei University College of Medicine in Seoul, Republic of Korea (IRB number: 4-2017-1093). The requirement for informed consent was waived for this study.

## Results

### Baseline characteristics

A total of 2070 patients were enrolled in this study. Table 1 shows the clinicopathologic characteristics of patients. Mean age at thyroidectomy was 45.6 ± 13.1 years old, and a female predominance was observed (84.6%). The median follow-up duration after surgery was 12.8 years (interquartile range: 11.7–14.1). A complete follow-up of survival status during the study period was available in 2006 (96.9%) patients; in the cohort, the loss to follow-up occurred for the first time 57 months after surgery. T1, T2, T3, and T4 classifications were noted in 69.4%, 13.4%, 11.3%, and 5.9% of patients, respectively. Also, regarding N classification, the frequency was 35.8% for N0, 42.7% for N1a, and 21.4% for N1b. Distant metastasis (M1 classification) was observed in 1.0% of patients. The frequency of each TNM stage was 83.8% for stage I, 13.2% for stage II, 2.6% for stage III, and 0.4% for stage IV. The main histologic type was papillary thyroid carcinoma (98.6%). The frequency of each comorbidity was as follows: 87 (4.2%) patients with malignancies other than DTC, 417 (20.1%) patients with HTN, 108 (5.2%) patients with DM, and 81 (3.9%) patients with chronic viral hepatitis. Coronary artery occlusive disease (including acute coronary syndrome), cerebrovascular accident, congestive heart failure, valvular heart disease, aortic dissection, atrial fibrillation, liver cirrhosis (all cases were associated with chronic viral hepatitis), asthma, chronic obstructive pulmonary disease, end-stage renal disease, connective tissue disease, dementia, and plegia had frequencies between 0.1% and 1.1%, respectively. Peripheral vascular disease, peptic ulcer disease, and AIDS were not observed as baseline comorbidities in this study cohort. A total of 607 patients (29.3%) had at least one comorbidity.

**Table 1:** Baseline characteristics of the patient cohort included in this study (N = 2070).

Characteristics	Values
Age at thyroidectomy, years^1^	45.6 ± 13.1
Age group^3^	
<45 years	998 (48.2%)
45–55 years	585 (28.3%)
55–65 years	330 (15.9%)
65–75 years	134 (6.5%)
≥75 years	23 (1.1%)
Follow up duration, years^2^	12.8 (11.7–14.1)
Gender^3^	
Female	1751 (84.6%)
Male	319 (15.4%)
T classification^3^	
T1	1436 (69.4%)
T2	278 (13.4%)
T3a	31 (1.5%)
T3b	202 (9.8%)
T4a	123 (5.9%)
T4b	0 (0.0%)
N classification^3^	
N0	742 (35.8%)
N1a	884 (42.7%)
N1b	444 (21.4%)
M classification^3^	
M0	2050 (99.0%)
M1	20 (1.0%)
TNM stage^3^	
I	1734 (83.8%)
II	273 (13.2%)
III	54 (2.6%)
IV	9 (0.4%)
Histology^3^	
Papillary thyroid carcinoma	2042 (98.6%)
Follicular thyroid carcinoma	27 (1.3%)
Hürthle cell carcinoma	1 (0.0%)
Preoperative comorbidities^3^	
Malignancies other than DTC	87 (4.2%)
Hypertension	417 (20.1%)
Coronary artery occlusive disease	15 (0.7%)
Peripheral vascular disease	0 (0.0%)
Cerebrovascular accident	15 (0.7%)
Congestive heart failure	4 (0.2%)
Valvular heart disease	10 (0.5%)
Aortic dissection	1 (0.0%)
Atrial fibrillation	9 (0.4%)
Diabetes mellitus (any)	108 (5.2%)
Diabetes mellitus with chronic complications	18 (0.9%)
Chronic viral hepatitis	81 (3.9%)
Liver cirrhosis	2 (0.1%)
Asthma	22 (1.1%)
Chronic obstructive pulmonary disease	8 (0.4%)
End-stage renal disease	16 (0.8%)
Connective tissue disease	4 (0.2%)
Dementia	2 (0.1%)
Hemiplegia, paraplegia, and quadriplegia	2 (0.1%)
Peptic ulcer disease	0 (0.0%)
Acquired immunodeficiency syndrome	0 (0.0%)
Total number of comorbidities in each patient^3^	
0	1463 (70.7%)
1	453 (21.9%)
2	121 (5.8%)
≥3	33 (1.6%)

Values are shown as ^1^mean ± standard deviation, ^2^median (interquartile range), or ^3^n (%). TNM, tumor-node-metastasis; DTC, differentiated thyroid carcinoma.

### Death from DTC, death from other causes, and survival

**Figure 1:**
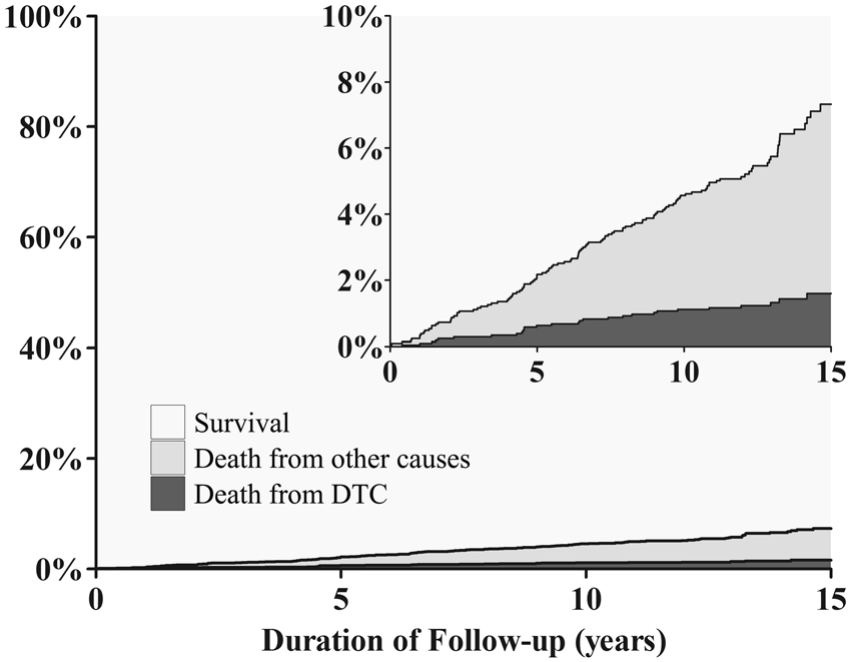
Cumulative incidence curves (CIC) of death from differentiated thyroid carcinoma (DTC) and death from other causes. The cumulative incidence functions are stacked, such the distances between the two curves represent the probabilities of death from other causes.

During the follow-up period, 28 cases of death from DTC and 95 cases of death from other causes were recorded. Non-thyroid malignancy (n = 37) and cardiovascular disease (n = 12) were the first and second most common causes of death other than DTC, respectively (Supplementary Table 1). The 15-year cumulative incidences of death from DTC, death from other causes, and overall death were 1.6%, 5.7%, and 7.3%, respectively (Fig. 1). Over the entire observation period, the cumulative incidence of death from other causes was higher than the cumulative incidence of death from DTC.

### Role of comorbidities in overall survival

**Figure 2:**
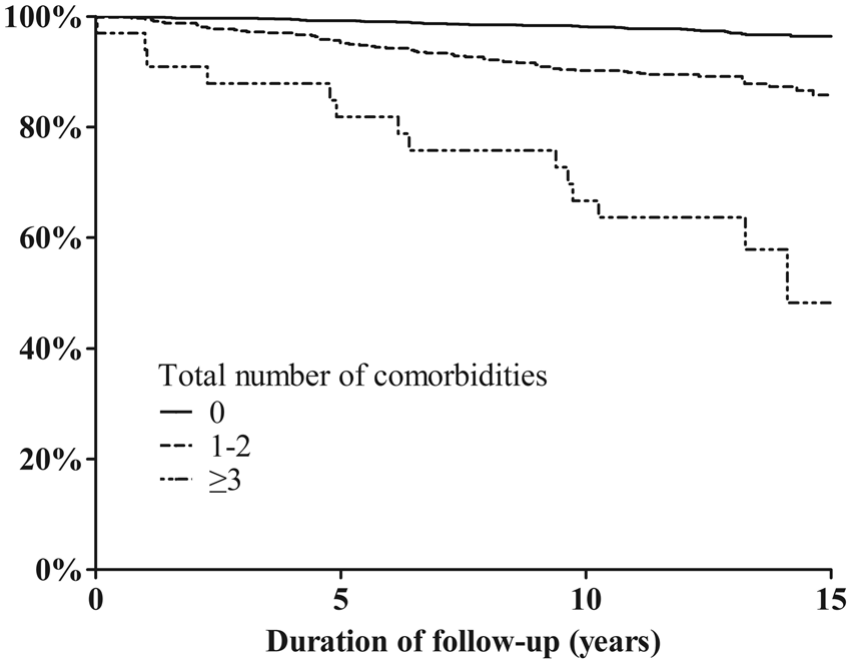
Overall survival curves of patients with thyroid cancer according to the number of comorbidities.

The Kaplan-Meier survival curves revealed differences in overall survival rates according to the number of comorbidities for each patient (log-rank, p < 0.01) (Fig. 2). The associations between T, N, and M classifications of DTC, the number of comorbidities, and overall survival were evaluated using a multivariable Cox regression model (Table 2). Higher T, N, and M classifications were associated with a shorter overall survival, as expected (T4 classification, HR = 3.50 [2.19–5.60]; N1b classification, HR = 1.69 [1.09–2.60]; and M1 classification, HR = 3.35 [1.49–7.52], with reference to T1, N0, and M0 classifications, respectively; all p < 0.05). However, the number of comorbidities was also independently associated with shorter overall survival after adjustment for age (<55, 55–65, and ≥65 years), sex, TNM classifications, and histologic types (number of comorbidities 1–2, HR = 1.95 [1.27–2.99]; number of comorbidities ≥3, HR = 5.33 [2.71–10.46]; both p < 0.01).

**Table 2:** Multivariable Cox proportional hazards model for the overall risk of death.

Variables	Number of deaths	Hazard ratio (95% CI)	*P* value
Age group			
<55 years (n = 1583)	37	1.00 (reference)	
55–65 years (n = 330)	34	3.15 (1.91–5.20)	<**0.01**
≥65 years (n = 157)	52	7.96 (4.86–13.02)	<**0.01**
Sex			
Female (n = 1751)	91		
Male (n = 319)	32	1.76 (1.17–2.65)	<**0.01**
T classification			
T1 (n = 1436)	50	1.00 (reference)	
T2 (n = 278)	22	1.76 (1.05–2.96)	**0.03**
T3 (n = 233)	19	1.61 (0.94–2.77)	0.08
T4 (n = 123)	32	3.50 (2.19–5.60)	<**0.01**
N classification			
N0 (n = 742)	48	1.00 (reference)	
N1a (n = 884)	28	0.80 (0.50–1.30)	0.37
N1b (n = 444)	47	1.69 (1.09–2.60)	**0.02**
M classification			
M0 (n = 2050)	116	1.00 (reference)	
M1 (n = 20)	7	3.35 (1.49–7.52)	<**0.01**
Histology			
PTC (n = 2042)	117	1.00 (reference)	
FTC or HCC (n = 28)	6	2.20 (0.91–5.32)	0.08
Number of comorbidities			
0 (n = 1463)	42	1.00 (reference)	
1–2 (n = 574)	67	1.95 (1.27–2.99)	<**0.01**
≥3 (n = 33)	14	5.33 (2.71–10.46)	<**0.01**

Hazard ratios for all-cause mortality according to tumor-node-metastasis (TNM) classification and the number of comorbidities were adjusted for age (<55, 55–65, and ≥65 years), sex, and histological type. CI, confidential interval; PTC, papillary thyroid carcinoma; FTC, Follicular thyroid carcinoma; HCC, Hürthle cell carcinoma. Significant hazard ratios are indicated in boldface font.

### Role of T, N, and M classifications of DTC and the number of comorbidities in death from DTC and death from other causes

The associations between T, N, and M classifications of DTC, the number of comorbidities, and the two types of death (death from DTC and death from other causes) were evaluated using Fine and Gray’s regression analyses (Table 3). Older age was associated with a high probability of both death from DTC and death from other causes (Age 55–65 years, SHR for death from DTC and death from other causes = 4.83 [1.40–16.61] and 2.69 [1.51–4.81], respectively; age >65 years, SHR for death from DTC and death from other causes = 14.15 [4.08–49.04] and 6.43 [3.55–1.64], respectively; all p < 0.05). Higher T, N, and M classifications were associated with a greater probability of death from DTC, as expected (T4 classification, SHR = 85.64 [9.88–742.4]; N1b classification, SHR = 9.07 [1.97–41.67]; and M1 classification, SHR = 5.70 [1.55–21.02], with reference to T1, N0, and M0 classifications, respectively; all p < 0.01), but were not associated with death from other causes. A higher number of comorbidities was associated with a high probability of death from other causes (number of comorbidities 1–2, SHR = 2.48 [1.46–4.20]; number of comorbidities ≥3, SHR = 9.41 [4.53–19.56]; both p < 0.01). Notably, a very high number of comorbidities (≥3) was also associated with extremely low probability of death from DTC (SHR = 6.81e-10 [1.87e-10–2.48e-9], p < 0.01). These results suggest that patients with many comorbidities had very low chances of dying from DTC because they were likely to die earlier from their comorbidities. We additionally performed the analysis using the Charlson comorbidity index instead of the number of comorbidities for generalization (Supplementary Table 2). The analyses showed similar results: patients with a score of four to five points on the Charlson comorbidity index had a high probability of death from other causes (SHR = 15.06 [4.24–53.53], p < 0.01) and a low probability of death from DTC (SHR = 2.15e-7 [3.85e-8–1.20e-6], p < 0.01).

**Table 3:** Multivariable competing risks regression model using the Fine and Gray method for death from DTC and death from other causes.

Variables	Death from DTC			Death from other causes		
	Number of deaths	Subhazard ratio (95% CI)	*P* value	Number of deaths	Subhazard ratio (95% CI)	*P* value
Age group						
<55 years (n = 1583)	6	1.00 (reference)		31	1.00 (reference)	
55–65 years (n = 330)	8	4.83 (1.40–16.61)	**0.01**	26	2.69 (1.51–4.81)	<**0.01**
≥65 years (n = 157)	14	14.15 (4.08–49.04)	<**0.01**	38	6.43 (3.55–11.64)	<**0.01**
Sex						
Female (n = 1751)	19	1.00 (reference)		72	1.00 (reference)	
Male (n = 319)	9	1.38 (0.54–3.52)	0.50	23	1.69 (1.06–2.69)	**0.03**
T classification						
T1 (n = 1436)	1	1.00 (reference)		49	1.00 (reference)	
T2 (n = 278)	3	11.84 (1.22–114.45)	**0.03**	19	1.70 (1.00–2.90)	0.05
T3 (n = 233)	4	17.98 (1.96–165.08)	**0.01**	15	1.28 (0.72–2.27)	0.41
T4 (n = 123)	20	85.64 (9.88–742.40)	<**0.01**	12	1.28 (0.63–2.57)	0.49
N classification						
N0 (n = 742)	2	1.00 (reference)		46	1.00 (reference)	
N1a (n = 884)	5	4.02 (0.83–19.56)	0.09	23	0.62 (0.37–1.05)	0.07
N1b (n = 444)	21	9.07 (1.97–41.67)	<**0.01**	26	1.02 (0.60–1.71)	0.95
M classification						
M0 (n = 2050)	24	1.00 (reference)		92	1.00 (reference)	
M1 (n = 20)	4	5.70 (1.55–21.02)	<**0.01**	3	1.60 (0.43–5.93)	0.48
Number of comorbidities						
0 (n = 1463)	13	1.00 (reference)		29	1.00 (reference)	
1–2 (n = 574)	15	0.88 (0.37–2.10)	0.77	52	2.48 (1.46–4.20)	<**0.01**
≥ 3 (n = 33)	0	6.81e-10 (1.87e-10–2.48e-9)	<**0.01**	14	9.41 (4.53–19.56)	<**0.01**

Subhazard ratios for death from DTC and death from other causes according to tumor-node-metastasis (TNM) classification and the number of comorbidities were adjusted for age (<55, 55–65, and ≥65 years) and sex. DTC, differentiated thyroid carcinoma; CI, confidential interval. Significant subhazard ratios are indicated in boldface font.

### Combined influence of TNM stage and the number of comorbidities on death from DTC, death from other causes, and overall death

**Figure 3:**
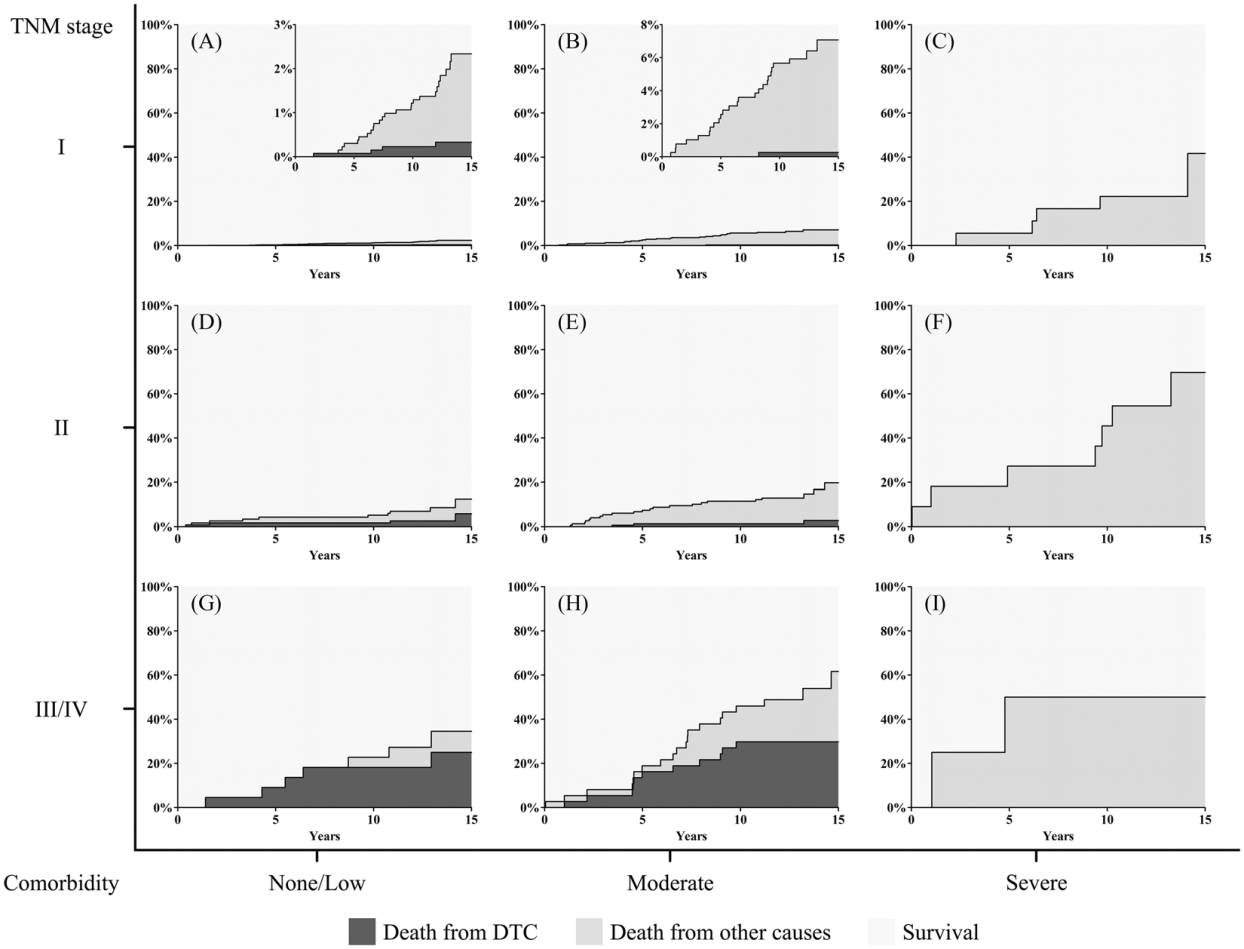
Cumulative incidence curves (CIC) of death from differentiated thyroid carcinoma (DTC) and death from other causes stratified by tumor-node-metastasis (TNM) stage and the number of comorbidities. The cumulative incidence functions are stacked, such the distances between the two curves represent the probabilities of death from other causes. (**A**) TNM stage I without comorbidities (n = 1326). (**B**) TNM stage I with 1 or 2 comorbidities (n = 390). (**C**) TNM stage I with ≥3 comorbidities (n = 18). (**D**) TNM stage II without comorbidities (n = 115). (**E**) TNM stage II with 1 or 2 comorbidities (n = 147). (**F**) TNM stage II with ≥3 comorbidities (n = 11). (**G**) TNM stage III or IV without comorbidities (n = 22). (**H**) TNM stage III or IV with 1 or 2 comorbidities (n = 37). (**I**) TNM stage III or IV with ≥3 comorbidities (n = 4).

To visualize the combined effects of DTC severity and the number of baseline comorbidities, we stratified patients according to the AJCC/UICC TNM stage and number of baseline comorbidities (Fig. 3). The frequency of each comorbidity and each cause of death, stratified by the TNM stage, is shown in Supplementary Tables 3 and 4. In this stratified plotting, the cumulative incidence of overall death increased when TNM stage or the number of comorbidities increased. In the groups without comorbidities, death from DTC accounted for a substantial proportion of the overall death, depending on the TNM stage (Fig. 3A,D,G; 15-year cumulative incidence of death from DTC/overall death = 0.3%/2.3% for TNM stage I, 5.9%/12.5% for TNM stage II, and 14.1%/18.1% for TNM stage III/IV). However, in the groups with 1 or 2 comorbidities, the relative proportion of death from DTC among the overall death was largely reduced (Fig. 3B,E,H; 15-year cumulative incidence of death from DTC/overall death = 0.3%/7.1% for TNM stage I, 2.9%/19.8% for TNM stage II, and 29.8%/61.6% for TNM stage III/IV). Moreover, death from DTC was not observed in the groups with comorbidities ≥3, in contrast to the high overall mortality rate in these groups (Fig. 3C,F,I; 15-year cumulative incidence of overall death = 41.7% for TNM stage I, 69.7% for TNM stage II, and 50.0% for TNM stage III/IV).

A higher number of comorbidities was associated with a higher cumulative incidence of death from other causes and overall death, regardless of the TNM stage of DTC. In particular, in the groups with TNM stage I DTC, almost all deaths were from other causes; hence, the cumulative incidences of overall death were largely dependent on the number of comorbidities (Fig. 3A–C).

## Discussion

In this study, we found that death from DTC accounted for only a fraction of the total deaths in patients who underwent surgery for DTC. Along with the severity of DTC, the baseline comorbidities at the time of surgery was also important factors in determining overall mortality. A higher number of comorbidities was mainly associated with a higher probability of death from other causes, but was also associated with a lower probability of death from DTC.

The management of cancer survivors (those living after a diagnosis of cancer) is becoming an increasingly important medical issue^2^. The number of cancer survivors is expected to increase steadily due to population growth, aging, and advances in treatment techniques for cancer^2,14^. Furthermore, the advance of diagnostic techniques and the increased implementation of neck ultrasonography, computed tomography, and fine needle aspiration are rapidly increasing the thyroid cancer incidence^15–18^. Because mortality from thyroid cancer was generally stable despite an increasing incidence of papillary thyroid cancers, many thyroid cancer survivors would ultimately die from causes other than the thyroid cancer itself^19^. Edwards *et al*. reported that comorbidities had a greater impact on non-cancer death and overall mortality in patients with local and regional disease compared to those with distant metastasis and that the role of comorbidities was more significant in less aggressive cancers, such as breast and prostate cancer, than in more aggressive cancers such as lung cancer^2^. Considering that DTC is a relatively indolent cancer, the inference that comorbidities would play an important role in DTC survivors is natural.

However, only a few studies have addressed the role of comorbidities in thyroid cancer to date. In 2006, Kuijpens *et al*. analyzed data from 417 patients with thyroid cancer including 353 patients with DTC, and reported that comorbidities did not independently affect the crude 5-year survival of patients with thyroid cancer^20^. However, because the study by Kuijpens *et al*. included medullary and anaplastic thyroid cancers and only had a small number of deaths, it was unclear whether the same results would be obtained if the study included a sufficient number of deaths in patients with DTC only. Recently, Karadaghy *et al*. reported that a staging system incorporating comorbidities could better predict the overall survival of patients with DTC than the AJCC/UICC staging system^21^. However, the causes of death were not considered in that study.

In our cohort, the 15-year cumulative incidence of overall death was 7.3%, but death from DTC accounted for only 1.6% (about one-fifth of overall deaths), despite excluding papillary thyroid microcarcinoma without metastases and NIFTP from the cohort. This relative proportion of cancer-specific death to overall death is comparable to that of localized breast or prostate cancer and far lower than that of regional cancer, distant cancer, and localized colorectal and lung cancer^2^. This finding suggests that medical problems other than DTC play a great role in the overall health of patients with DTC.

In subsequent analyses, we found that baseline comorbidities lower overall survival, independent of the TNM classification of DTC. This is in agreement with the report by Karadaghy *et al*., in which the severity of comorbidities had a predictive role for the overall survival of patients with DTC^21^. A higher number of comorbidities was associated with a higher probability of death from causes other than DTC, but did not increase deaths from DTC itself. Furthermore, a very high number of comorbidities (≥3) lowered the likelihood of death from DTC. In a situation with competing risks, the probability of one outcome is influenced by the risk of the other outcomes^11,12,22^. In a recent controversy about the increased mortality rate from thyroid cancer in Japan, Davies *et al*. suggested that this phenomenon may be due to long life expectancies and lower mortality rates from other causes^23^. Inversely, our results demonstrate that patients with multiple comorbidities may have a low likelihood of dying from DTC because they are more likely to die earlier from their comorbidities.

The roles of comorbidities and deaths from other causes were more pronounced in patients with a lower stage of DTC. It is notable that almost all observed deaths in patients with stage I DTC were from causes other than DTC. Death from other causes also accounted for most of the overall deaths in patients with stage II DTC. Thus, overall mortality in patients with stage I/II DTC was largely dependent on the number of comorbidities. These results suggest that special attention should be paid to the comorbidities of patients with early-stage DTC that account for the majority of all DTC cases.

Older age is a well-recognized prognostic determinant of cancer-specific death in DTC^24,25^, which was also observed in our study. However, older age was also associated with a greater probability of a death from other causes. Therefore, in older patients, a balanced clinical approach is needed to consider the possibility of death from both DTC and other causes.

There were several limitations to this study. Because this was a single-center study, there could be selection bias depending on the patients’ visiting patterns or the treatment patterns. Because we only included patients who underwent thyroidectomy, our results should not be directly generalized to patients under active surveillance. Because most patients included in our study had papillary thyroid carcinoma, the study results should be interpreted with caution in patients with other types of DTC. The duration of each comorbidity was not considered, and each category of comorbidities included many different conditions with different mortality risks. However, our results were also supported by additional analysis using Charlson comorbidity index. Changes in comorbidities during the follow-up period were not considered; thus, we could not confirm the effects of improvement, exacerbation, or emergence of comorbidities or the effects of treatments for comorbidities.

To the best of our knowledge, this is the first study to demonstrate that comorbidities are important factors for determining not only the overall survival but also cause of death in patients with DTC, with a large cohort and long term follow-up duration. Our study reminds clinicians that comorbidities and mortality risks that are not directly related with thyroid cancer could be important medical issues in some thyroid cancer patients. Evaluating and considering the comorbidities of a patient with DTC will help the physician to better understand a patient’s overall health status and guide clinical decisions regarding follow-up planning for postoperative patients with DTC.

### Précis

Many patients with differentiated thyroid carcinoma (DTC) ultimately die from comorbidities rather than DTC itself. Multiple comorbidities lower the probability of dying from DTC in a competitive manner.

## Supplementary information

Supplementary Information

## Data Availability

The datasets generated during and/or analyzed during the current study are not publicly available due to protection of patient information but are available from the corresponding author on reasonable request.
