# Supplementary material for: The relationship of comorbidities to mortality and cause of death in patients with differentiated thyroid carcinoma
Source: Sci Rep. 2019 Aug 7;9:11435. doi: 10.1038/s41598-019-47898-8 (PMC6685995; doi:10.1038/s41598-019-47898-8)
Supplement: Supplementary file 1 — Supplementary Information [file 41598_2019_47898_MOESM1_ESM.docx]

**Supplementary Information to:**

**The relationship of comorbidities to mortality and cause of death in patients with differentiated thyroid carcinoma**

**Young Ki Lee MD,^1,2^ Namki Hong MD,^1^ Se Hee Park MD,^3^ Dong Yeob Shin MD, PhD,^1^ Cho Rok Lee MD,^4^ Sang-Wook Kang MD, PhD,^4^ Jandee Lee MD, PhD,^4^ Jong Ju Jeong MD,^4^ Kee-Hyun Nam MD, PhD,^4^ Woong Youn Chung MD, PhD,^4^ Eun Jig Lee MD, PhD^1^**

^1^Division of Endocrinology and Metabolism, Department of Internal Medicine, Yonsei University College of Medicine, Seoul 03722, Republic of Korea

^2^Center for Thyroid Cancer, National Cancer center, Goyang 10408, Republic of Korea

^3^Division of Endocrinology and Metabolism, Department of Internal Medicine, National Health Insurance Service Ilsan Hospital, Goyang 10444, Republic of Korea

^4^Division of Thyroid and Endocrine Surgery, Department of Surgery, Yonsei University College of Medicine, Seoul 03722, Republic of Korea.

**Corresponding author:** Dong Yeob Shin, MD, PhD

Division of Endocrinology and Metabolism, Department of Internal Medicine, Yonsei University College of Medicine, 50-1 Yonsei-ro, Seodaemun-gu, Seoul 03722, Republic of Korea

E-mail: shindongyi@yuhs.ac;

Tel: +82-2-2228-2261; Fax: +82-2-393-6884

Supplementary Table 1. Specific causes of death other than differentiated thyroid cancer

| Specific causes of death | Number of patients |
| --- | --- |
| Non-thyroidal malignancy | 37 |
| Lung cancer | 11 |
| Breast cancer | 4 |
| Stomach cancer | 3 |
| Acute myeloid leukemia | 2 |
| Colon cancer | 2 |
| Esophageal cancer | 2 |
| Hepatocellular carcinoma | 2 |
| Ovarian cancer | 2 |
| Pancreas cancer | 2 |
| Gallbladder cancer | 1 |
| Malignant phyllodes tumor | 1 |
| Multiple myeloma | 1 |
| Prostate cancer | 1 |
| Rabdomyosarcoma | 1 |
| Renal cellular carcinoma | 1 |
| Ureter cancer | 1 |
| Cardiovascular disease | 12 |
| Chronic lung disease | 3 |
| Traffic accident | 3 |
| Suicide | 3 |
| Infection | 2 |
| Senile death | 2 |
| End-stage renal disease | 1 |
| Amyotrophic lateral sclerosis | 1 |
| Alzheimer's disease | 1 |
| Diabetes mellitus | 1 |
| Unknown (no evidence of residual thyroid cancer)^1^ | 29 |

^1^“Unknown” indicates patients who had neither evidence of residual thyroid cancer nor other proven causes of death.

Supplementary Table 2. Multivariable competing risks regression model using the Fine and Gray method for death from DTC and death from other causes.

| Variables | Death from DTC | | | Death from other causes | | |
| --- | --- | --- | --- | --- | --- | --- |
|  | Number of deaths | Subhazard ratio (95% CI) | *P* value | Number of deaths | Subhazard ratio (95% CI) | *P* value |
| Age group |  |  |  |  |  |  |
| <55 years (n = 1583) | 6 | 1.00 (reference) |  | 31 | 1.00 (reference) |  |
| 55–65 years (n = 330) | 8 | 4.86 (1.41–16.80) | **0.01** | 26 | 3.09 (1.77–5.38) | **<0.01** |
| ≥65 years (n = 157) | 14 | 13.63 (4.15–44.75) | **<0.01** | 38 | 8.42 (4.84–14.65) | **<0.01** |
| Sex |  |  |  |  |  |  |
| Female (n = 1751) | 19 | 1.00 (reference) |  | 72 | 1.00 (reference) |  |
| Male (n = 319) | 9 | 1.35 (0.52–3.55) | 0.54 | 23 | 1.52 (0.94–2.45) | 0.09 |
| T classification |  |  |  |  |  |  |
| T1 (n = 1436) | 1 | 1.00 (reference) |  | 49 | 1.00 (reference) |  |
| T2 (n = 278) | 3 | 11.98 (1.24–116.06) | **0.03** | 19 | 1.86 (1.09–3.18) | **0.02** |
| T3 (n = 233) | 4 | 18.21 (1.97–168.50) | **0.01** | 15 | 1.45 (0.81–2.59) | 0.22 |
| T4 (n = 123) | 20 | 82.02 (9.50–707.80) | **<0.01** | 12 | 1.45 (0.71–2.96) | 0.31 |
| N classification |  |  |  |  |  |  |
| N0 (n = 742) | 2 | 1.00 (reference) |  | 46 | 1.00 (reference) |  |
| N1a (n = 884) | 5 | 4.12 (0.83–20.56) | 0.08 | 23 | 0.67 (0.39–1.14) | 0.14 |
| N1b (n = 444) | 21 | 10.39 (2.22–48.67) | **<0.01** | 26 | 0.99 (0.57–1.70) | 0.96 |
| M classification |  |  |  |  |  |  |
| M0 (n = 2050) | 24 | 1.00 (reference) |  | 92 | 1.00 (reference) |  |
| M1 (n = 20) | 4 | 5.61 (1.40–22.46) | **0.02** | 3 | 1.72 (0.39–7.64) | 0.48 |
| Charlson comorbidity index^1^ |  |  |  |  |  |  |
| 0 point (n = 1781) | 24 | 1.00 (reference) |  | 50 | 1.00 (reference) |  |
| 1–3 points (n = 280) | 4 | 0.55 (0.18–1.66) | 0.29 | 40 | 3.09 (1.94–4.91) | **<0.01** |
| ≥4 points (n = 9) | 0 | 2.15e-7 (3.85e-8–1.20e-6) | **<0.01** | 5 | 15.06 (4.24–53.53) | **<0.01** |

Subhazard ratios for death from DTC and death from other causes according to tumor-node-metastasis (TNM) classification and the Charlson comorbidity index were adjusted for age (<55, 55–65, and ≥65 years) and sex. DTC, differentiated thyroid carcinoma; CI, confidential interval. ^1^Age and thyroid cancer were not used for calculating the Charlson comorbidity index. Significant subhazard ratios are indicated in boldface font.

Supplementary Table 3. Comorbidities stratified by the tumor-node-metastasis stage of differentiated thyroid carcinoma

| Characteristics | Tumor-node-metastasis stage of DTC | | | |
| --- | --- | --- | --- | --- |
|  | I (n = 1734) | II (n = 273) | III (n = 54) | IV (n = 9) |
| Preoperative comorbidities |  |  |  |  |
| Malignancies other than DTC | 63 (3.6%) | 20 (7.3%) | 4 (7.4%) | 0 (0.0%) |
| Hypertension | 258 (14.9%) | 125 (45.8%) | 27 (50.0%) | 7 (77.8%) |
| Coronary artery occlusive disease | 7 (0.4%) | 5 (1.8%) | 2 (3.7%) | 1 (11.1%) |
| Cerebrovascular accident | 9 (0.5%) | 4 (1.5%) | 2 (3.7%) | 0 (0.0%) |
| Congestive heart failure | 2 (0.1%) | 2 (0.7%) | 0 (0.0%) | 0 (0.0%) |
| Valvular heart disease | 4 (0.2%) | 5 (1.8%) | 1 (1.9%) | 0 (0.0%) |
| Aortic dissection | 1 (0.1%) | 0 (0.0%) | 0 (0.0%) | 0 (0.0%) |
| Atrial fibrillation | 6 (0.3%) | 1 (0.4%) | 2 (3.7%) | 0 (0.0%) |
| Diabetes mellitus (any) | 62 (3.6%) | 39 (14.3%) | 6 (11.1%) | 1 (11.1%) |
| Diabetes mellitus with chronic complications | 9 (0.5%) | 7 (2.6%) | 2 (3.7%) | 0 (0.0%) |
| Chronic viral hepatitis | 67 (3.9%) | 12 (4.4%) | 2 (3.7%) | 0 (0.0%) |
| Liver cirrhosis | 1 (0.1%) | 1 (0.4%) | 0 (0.0%) | 0 (0.0%) |
| Asthma | 16 (0.9%) | 5 (1.8%) | 0 (0.0%) | 1 (11.1%) |
| Chronic obstructive pulmonary disease | 4 (0.2%) | 3 (1.1%) | 0 (0.0%) | 1 (11.1%) |
| End-stage renal disease | 16 (0.9%) | 0 (0.0%) | 0 (0.0%) | 0 (0.0%) |
| Connective tissue disease | 4 (0.2%) | 0 (0.0%) | 0 (0.0%) | 0 (0.0%) |
| Dementia | 0 (0.0%) | 2 (0.7%) | 0 (0.0%) | 0 (0.0%) |
| Hemiplegia, paraplegia, and quadriplegia | 1 (0.1%) | 0 (0.0%) | 1 (1.9%) | 0 (0.0%) |
| Total number of comorbidities in each patient |  |  |  |  |
| 0 | 1326 (76.5%) | 115 (42.1%) | 21 (38.9%) | 1 (11.1%) |
| 1 | 319 (18.4%) | 105 (38.5%) | 22 (40.7%) | 7 (77.8%) |
| 2 | 71 (4.1%) | 42 (15.4%) | 8 (14.8%) | 0 (0.0%) |
| ≥3 | 18 (1.0%) | 11 (4.0%) | 3 (5.6%) | 1 (11.1%) |

DTC, differentiated thyroid carcinoma. Values are shown as n (%).

Supplementary Table 4. Specific causes of death stratified by the tumor-node-metastasis stage of differentiated thyroid carcinoma

| Specific causes of death | Tumor-node-metastasis stage of DTC | | | |
| --- | --- | --- | --- | --- |
|  | I (n = 1734) | II (n = 273) | III (n = 54) | IV (n = 9) |
| Thyroid cancer | 5 | 7 | 13 | 3 |
| Non-thyroidal malignancy | 19 | 14 | 4 | 0 |
| Lung cancer | 5 | 4 | 2 | 0 |
| Breast cancer | 3 | 1 | 0 | 0 |
| Stomach cancer | 3 | 0 | 0 | 0 |
| Acute myeloid leukemia | 1 | 1 | 0 | 0 |
| Colon cancer | 0 | 1 | 1 | 0 |
| Esophageal cancer | 1 | 1 | 0 | 0 |
| Hepatocellular carcinoma | 2 | 0 | 0 | 0 |
| Ovarian cancer | 0 | 1 | 1 | 0 |
| Pancreas cancer | 0 | 2 | 0 | 0 |
| Gallbladder cancer | 1 | 0 | 0 | 0 |
| Malignant phyllodes tumor | 1 | 0 | 0 | 0 |
| Multiple myeloma | 1 | 0 | 0 | 0 |
| Prostate cancer | 0 | 1 | 0 | 0 |
| Rabdomyosarcoma | 1 | 0 | 0 | 0 |
| Renal cellular carcinoma | 0 | 1 | 0 | 0 |
| Ureter cancer | 0 | 1 | 0 | 0 |
| Cardiovascular disease | 7 | 4 | 0 | 1 |
| Chronic lung disease | 1 | 1 | 0 | 1 |
| Traffic accident | 1 | 1 | 1 | 0 |
| Suicide | 2 | 1 | 0 | 0 |
| Infection | 1 | 0 | 1 | 0 |
| Senile death | 0 | 1 | 0 | 1 |
| End-stage renal disease | 1 | 0 | 0 | 0 |
| Amyotrophic lateral sclerosis | 0 | 1 | 0 | 0 |
| Alzheimer's disease | 0 | 0 | 1 | 0 |
| Diabetes mellitus | 1 | 0 | 0 | 0 |
| Unknown  (no evidence of residual thyroid cancer)^1^ | 17 | 9 | 3 | 0 |

The number of deaths for each cause was presented. ^1^“Unknown” indicates patients who had neither evidence of residual thyroid cancer nor other proven causes of death.
